# Overexpression of Kpnβ1 and Kpnα2 Importin Proteins in Cancer Derives from Deregulated E2F Activity

**DOI:** 10.1371/journal.pone.0027723

**Published:** 2011-11-18

**Authors:** Pauline J. van der Watt, Ellen Ngarande, Virna D. Leaner

**Affiliations:** Division of Medical Biochemistry, Faculty of Health Sciences, Institute of Infectious Disease and Molecular Medicine, University of Cape Town, Cape Town, South Africa; Roswell Park Cancer Institute, United States of America

## Abstract

The Karyopherin superfamily comprises nuclear transport proteins, involved in the shuttling of certain cargo proteins into and out of the nucleus. Karyopherin β1 (Kpnβ1) and Karyopherin α2 (Kpnα2) are importin proteins, which work in concert to transport their cargo into the nucleus. We previously identified increased expression of Kpnβ1 and Kpnα2 in cervical tumours compared to normal epithelium and in transformed cells compared to their normal counterparts. This study therefore aimed to identify the transcription regulatory mechanisms associated with high Kpnβ1 and Kpnα2 levels in cancer cells. Kpnβ1 (−2013 to +100) and Kpnα2 (−1900 to +69) promoter fragments were separately cloned into the reporter vector, pGL3-basic, and luciferase assays revealed both as significantly more active in cancer and transformed cells compared to normal. A series of deletion constructs identified the −637 to −271 Kpnβ1 and −180 to −24 Kpnα2 promoter regions as responsible for the differential promoter activity, and a number of highly conserved E2F binding sites were identified within these regions. Mutation analysis confirmed the requirement of E2F sites for promoter activity, and ChIP analysis confirmed E2F2/Dp1 binding to the Kpnβ1 and Kpnα2 promoters *in vivo*. Dp1 inhibition resulted in decreased levels of the respective proteins, confirming the role of E2F in the overexpression of Kpnβ1 and Kpnα2 proteins in cancer. E2F activity is known to be deregulated in cervical cancer cells due to the inhibition of its repressor, Rb, by HPV E7. The inhibition of E7 using siRNA resulted in decreased Kpnβ1 and Kpnα2 promoter activities, as did the overexpression of Rb. In conclusion, this study is a first to show that elevated Kpnβ1 and Kpnα2 expression in cancer cells correlates with altered transcriptional regulation associated with deregulated E2F/Rb activities

## Introduction

Karyopherin proteins are soluble nuclear transport receptors that function in transporting cargo proteins and certain RNAs into and out of the cell nucleus, via the nuclear pore complex (NPC) [1]. Karyopherins may act as importins of exportins, depending of their direction of transport. Karyopherin beta 1 (Kpnβ1, also known as Importin β or p97) is an importin protein that plays a key role in the nuclear import process. Nuclear import via Kpnβ1 can occur either by Kpnβ1 acting as an autonomous nuclear transport receptor, or through its association with an adaptor protein, like Karyopherin alpha (Kpnα, also known as Importin α), in which case the import process is known as classical nuclear import [2]. In the classical nuclear import pathway, the nuclear localisation sequence (NLS) present in the cargo is recognised and bound by the adaptor protein, Kpnα, which then forms a trimer complex with Kpnβ1. The cargo:Kpnα:Kpnβ1 complex is transported across the nuclear pore complex into the nucleus, where upon it is dissociated by the binding of RanGTP. There are six Kpnα isoforms (Kpnα1-6), which have been determined to have preferential binding to specific substrates [3]. The functioning of all six isoforms thus broadens the substrate range carried across the NPC.

We have recently shown that the expression of Kpnβ1 and the Kpnα protein, Kpnα2, is elevated in cervical cancer and transformed cells at both the mRNA and protein levels [4]. We found too that inhibition of Kpnβ1 expression in cervical cancer cells leads to cell death via apoptosis, suggesting that cervical cancer cells become functionally dependent on Kpnβ1 overexpression, highlighting the importance of Kpnβ1 upregulation in maintaining cancer biology. In our study Kpnα2 inhibition had no effect on cervical cancer cell viability [4]; it is possible that its inhibition resulted in functional compensation by other Kpnα family members. Noetzel et al. [5] showed that breast cancer cells underwent apoptotic cell death when Kpnα2 was inhibited [5], suggesting that in some cell lines it might be functionally redundant, while in others it is functionally relevant. In addition, many recent studies have identified high Kpnα2 in tumour tissue, suggesting that the upregulation of Kpnα2 associates with cancer development. Such cancers include melanoma [6], breast cancer [7]; [8], oesophageal cancer [9], ovarian cancer [10], non-small cell lung cancer [11], prostate cancer [12], bladder cancer [13] and liver cancer [14]. Interestingly, Wang et al. [11] showed that Kpnα2 is secreted into the serum, suggesting it has potential clinical usefulness as a cancer biomarker. In addition, several studies have reported that high Kpnα2 expression associates with poor patient survival [6]–[10]; [12]; [13], suggesting a potential prognostic role for Kpnα2.

While the overexpression of Kpnβ1 and Kpnα2 in cancer cells appear to be significant events, little is known regarding their transcriptional regulation and the factors that control their expression and lead to their upregulation in cancer cells. In this study, therefore, we cloned and analysed the Kpnβ1 and Kpnα2 promoters, and identified an important role for the transcription factor, E2F, in the induction of Kpnβ1 and Kpnα2 expression in cancer cells.

## Results

### Increased Kpnβ1 and Kpnα2 protein expression in transformed and cancer cells correlates with increased promoter activity

**Figure 1:**
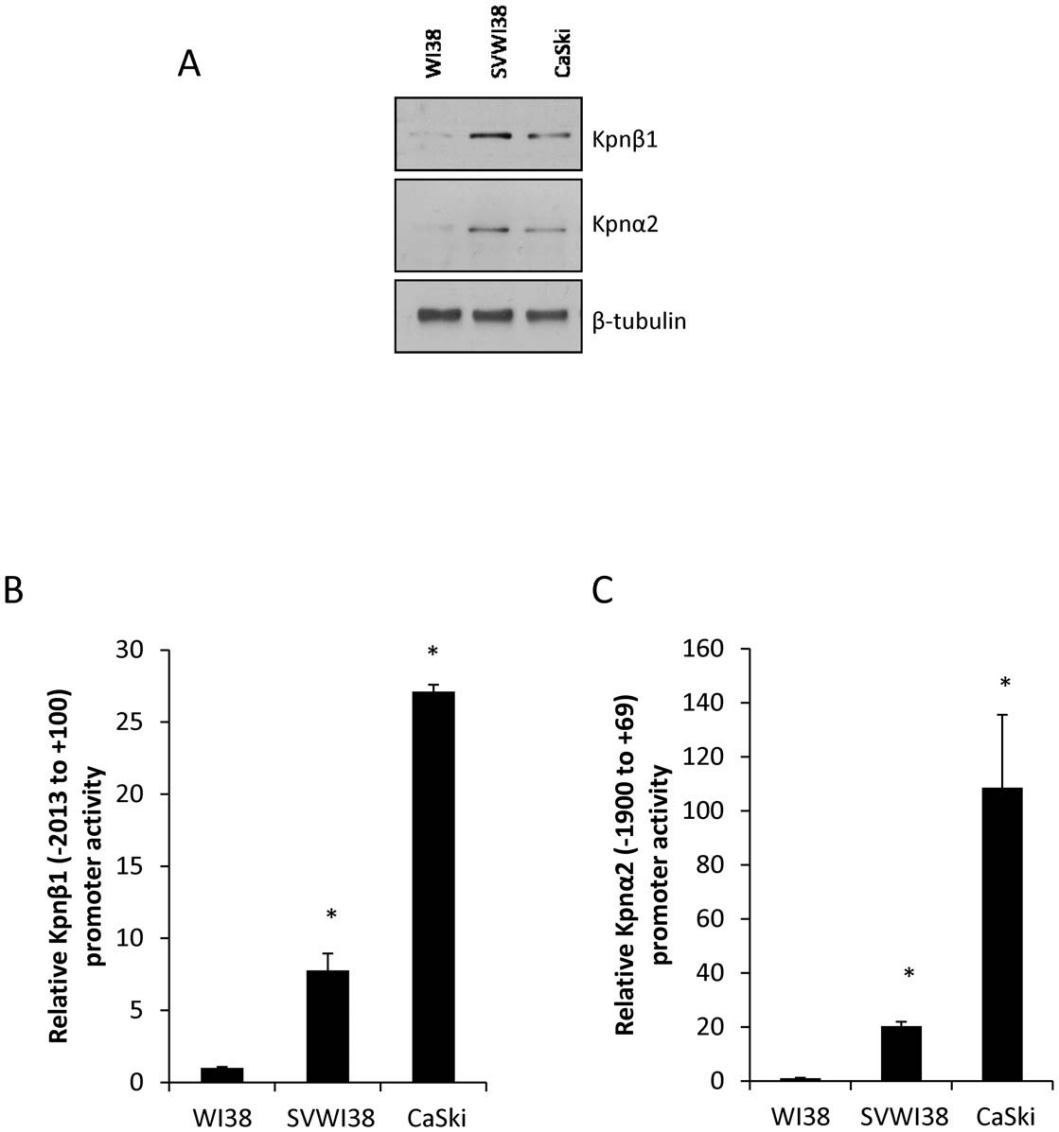
Kpnβ1 and Kpnα2 protein levels in normal, transformed and cancer cells correlate with respective promoter activities. A. Western Blot analysis of Kpnβ1 and Kpnα2 protein levels in normal WI38, transformed SVWI38, and cervical cancer CaSki cells reveals increased expression in the cancer and transformed cells. B, C. −2013 to +100 Kpnβ1 promoter activity (B) and −1900 to +69 Kpnα2 promoter activity (C) was measured in cell lysates prepared from transfected WI38, SVWI38 and CaSki cells, and was observed to be significantly higher in the transformed and cancer cells compared to the normal cells (*p<0.05). TK-Renilla-Luc was used as a control for transfection efficiency and luciferase activity is expressed relative to Renilla luciferase. Results shown are the mean ± SD of experiments performed in triplicate and repeated at least two times.

Based on our previous findings showing elevated Kpnβ1 and Kpnα2 expression in cervical cancer cells and transformed cells compared to normal, we investigated Kpnβ1 and Kpnα2 protein expression in a representative normal, transformed and cervical cancer cell line. These included the normal fibroblast cell line, WI38, the SV40-transformed fibroblast cell line, SVWI38, and the cervical cancer cell line, CaSki. Western Blot analysis revealed elevated Kpnβ1 and Kpnα2 expression in the transformed and cancer cells, compared to the normal WI38 cells (Fig. 1A). To determine the transcriptional regulatory mechanisms that associate with elevated Kpnβ1 and Kpnα2 expression in cancer and transformed cells, an approximate 2 Kb region upstream of the transcription start site of the Kpnβ1 and Kpnα2 genes, together with approximately 100 bp of their 5′ untranslated regions (downstream from the transcription start site), were separately cloned into pGL3-Basic for promoter analysis. The −2013 to +100 Kpnβ1 and −1990 to +69 Kpnα2 promoter constructs both showed significantly higher activity in the transformed and cancer cells compared to the normal WI38 cells (Fig. 1B and C).

### Differential Kpnβ1 and Kpnα2 promoter activities in normal, transformed and cancer cells derive from the −637 to −271 and −180 to −24 regions of the Kpnβ1 and Kpnα2 promoters, respectively

**Figure 2:**
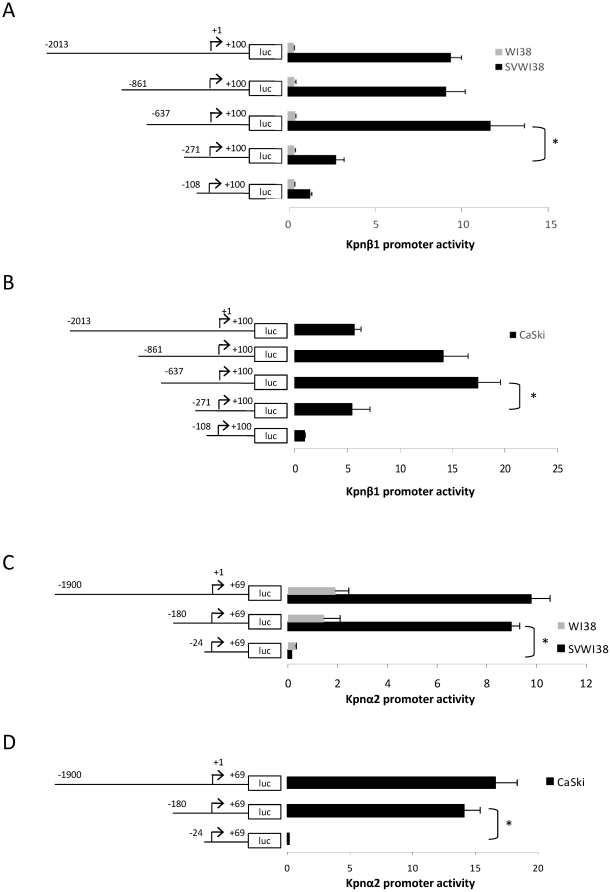
Activity of Kpnβ1 and Kpnα2 promoter deletion constructs in WI38, SVWI38 and CaSki cells. A. Luciferase reporter constructs containing deleted fragments of the human Kpnβ1 promoter were transiently transfected into WI38 and SVWI38 cells, respectively. Luciferase activity was significantly higher in the SVWI38 cells compared to normal WI38 cells for all constructs tested. The −637 to +100 Kpnβ1 promoter fragment contained significantly more promoter activity than the −271 to +100 fragment in transformed cells (*p<0.05). B. Activity of Kpnβ1 promoter deletion constructs in CaSki cells. C. Luciferase reporter constructs containing deleted fragments of the human Kpnα2 promoter were transiently transfected into WI38 and SVWI38 cells, respectively. Deletion of the −180 to −24 promoter region resulted in significantly diminished promoter activity. D. Activity of Kpnα2 promoter deletion constructs in CaSki cells.

In order to determine the regions responsible for the differential activity of Kpnβ1 and Kpnα2 promoters in normal and transformed/cancer cells, a series of deletion constructs were generated and assayed for activity in WI38, SVWI38 and CaSki cells. All deletion constructs of the Kpnβ1 promoter displayed significantly more activity in SVWI38 cells compared to WI38 cells (Fig. 2A). Notably in SVWI38 cells sequential deletion of the −2013 to −637 region did not alter promoter activity, however, deletion of the −637 to −271 region significantly diminished Kpnβ1 promoter activity (by approximately five-fold) (Fig. 2A). In WI38 cells, deletion of this region had little effect on promoter activity. In CaSki cells similar observations were made to those in SVWI38 cells, where deletion of the region from −637 to −271 significantly altered Kpnβ1 promoter activity (Fig. 2B). These results suggest that important functional elements, necessary for high Kpnβ1 promoter activity in transformed and cervical cancer cells, are likely to reside in the −637 to −271 region of the Kpnβ1 promoter. With regards to Kpnα2, deletion of the region from 1900 to −180 had little effect on Kpnα2 promoter activity in any of the cell lines (Fig. 2C, D), suggesting that the region upstream of −180 in the Kpnα2 promoter does not confer transcriptional activity. However, deletion of the region from −180 to −24 resulted in significantly diminished promoter activity, almost to the level of the empty vector, with the decrease in activity being more pronounced in SVWI38 and CaSki cells, compared to WI38 (Fig. 2C, D). This suggests that important functional elements necessary for high basal Kpnα2 promoter activity in transformed and cervical cancer cells reside in the −180 to −24 region of the promoter.

### The Kpnβ1 and Kpnα2 promoters contain functional E2F sites

**Figure 3:**
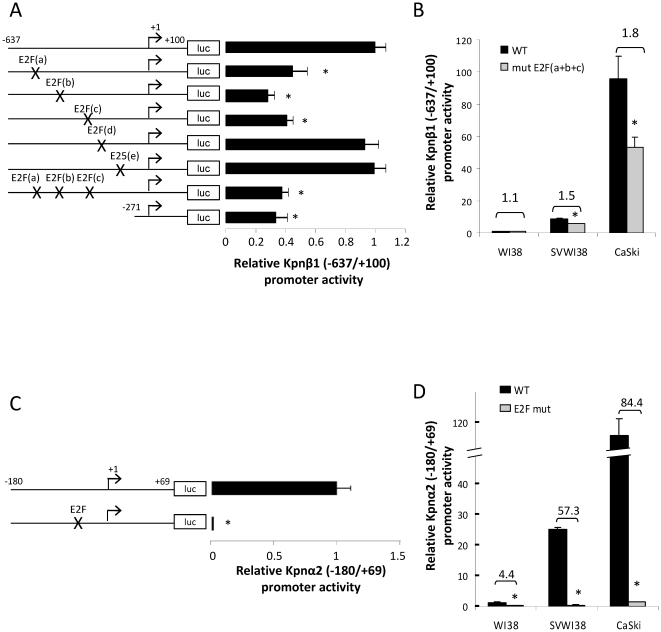
Functional E2F sites in the Kpnβ1 (−637 to −271) and Kpnα2 (−180 to −24) promoter regions contribute to high promoter activity in transformed and cancer cells. A. Mutation of the five putative E2F sites in the Kpnβ1 (−637 to +100) promoter was carried out by site-directed mutagenesis. Mutation of the three distal E2F sites lead to a significant decrease in Kpnβ1 (−637 to +100) promoter activity, as indicated by luciferase assays. Mutation of the three functional E2F sites simultaneously did not lead to any further decrease in promoter activity. B. Mutation of the three E2F sites in SVWI38 and CaSki cells had a more profound impact on Kpnβ1 promoter activity compared to that in WI38 cells (numbers indicate fold repression). C. Mutation of the single E2F site in the Kpnα2 promoter completely abolished promoter activity. D. Mutation of the E2F site in SVWI38 and CaSki cells had a more profound impact on Kpnα2 promoter activity compared to that in WI38 cells. Experiments are shown as the mean ± SE of experiments in quadruplicate performed at least two times (* p<0.05).

A bioinformatic analysis of the regions −637 to −271 of the Kpnβ1 promoter and −180 to −24 of the Kpnα2 promoter (using MatInspector and Conreal software programmes) identified a number of putative binding sites for E2F. Deregulated E2F activity is known to play a key role in cancer development; hence the functionality of these putative E2F sites was investigated. Five putative E2F binding sites were identified in the Kpnβ1 (−637 to −271) promoter region, and using site-directed mutagenesis (and mutating at least three bases within the consensus binding sites), mutant constructs were generated. Three E2F sites were found to be functional, as their mutation resulted in significantly decreased Kpnβ1 promoter activity (Fig. 3A). This included the putative sites labelled as E2F(a), E2F(b) and E2F(c). Interestingly, mutation of these three sites simultaneously did not confer any further decrease in promoter activity, compared to mutation of the E2F sites individually, suggesting that they may work in concert to regulate Kpnβ1 gene expression (Fig. 3A). Of note, too, was the finding that mutation of the E2F sites resulted in promoter activity comparable to that of the −271 to +100 construct, suggesting that it is the E2F sites that are responsible for the increase in promoter activity in the region from −271 to −637. Mutation of the three E2F sites in the Kpnβ1 promoter had a more pronounced effect on promoter activity in SVWI38 and CaSki cells compared to that in WI38 cells. These findings suggest that there is increased reliance on the E2F sites in transformed and cancer cells, compared to normal (Fig. 3B).

Bioinformatic analysis of the Kpnα2 promoter, on the other hand, revealed the presence of only one putative E2F site in the −180 to −24 region. Interestingly, unlike the E2F sites in the Kpnβ1 promoter, mutation of this single E2F site completely abolished Kpnα2 promoter activity (Fig. 3C). Mutation of the E2F site in SVWI38 and CaSki cells resulted in 84- and 57-fold repression of promoter activity, respectively, while its mutation in WI38 cells resulted in only 4-fold repression of the promoter (Fig. 3D).

Together, these results suggest that the Kpnβ1 promoter contains E2F sites that act to enhance promoter activity, while the Kpnα2 promoter contains an E2F site that is involved in basal promoter activation. In addition, E2F activation of both promoters is more pronounced in transformed and cancer cells, compared to normal.

### E2F/Dp1 heterodimers bind and activate the Kpnβ1 and Kpnα2 promoters *in vivo*

**Figure 4:**
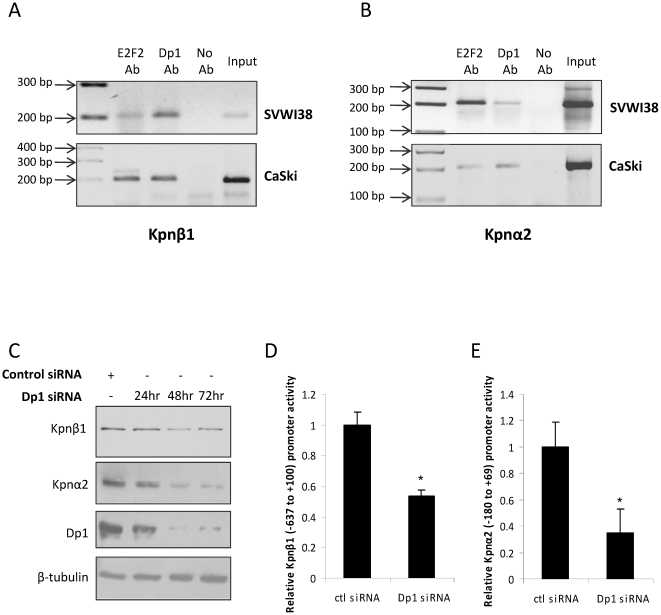
E2F/DP1 binds and activates the Kpnβ1 and Kpnα2 promoters *in vivo*. A, B. Samples of sonicated chromatin were immunoprecipitated with an α-E2F2 antibody, an α-DP1 antibody, or no antibody, as indicated. DNA isolated from immunoprecipitated material was amplified by PCR using primers spanning the E2F sites present in the Kpnβ1 (A) or Kpnα2 (B) promoters. Amplified fragments were analysed by electrophoresis on a 2% agarose gel. Experiments were performed in both SVWI38 and CaSki cell lines. C. Western Blot showing Kpnβ1 and Kpnα2 protein levels after Dp1 inhibition using siRNA. β-tubulin was used as a control for protein loading. D, E. Kpnβ1 and Kpnα2 promoter activities after Dp1 inhibition. Results shown are the mean ± SE of experiments in quadruplicate performed at least two times (* p<0.05).

E2F functions as a heterodimeric transcription factor composed of two subunits: an E2F family member and a Dp family member. Although there are multiple E2F family members that are thought to bind the same consensus sequence (5′-TTTSSCGS-3′; S refers to C or G), E2F1, E2F2 and E2F3 have been shown to contain strong oncogenic capacity [15], hence binding of E2F to the Kpnβ1 and Kpnα2 promoters was investigated using an antibody against E2F2 that cross-reacts with E2F1, E2F2 and E2F3. The Dp protein family comprises proteins Dp1-3, with Dp1 having been previously linked to cancer. To assess E2F2/Dp1 binding *in vivo*, ChIP assays were performed using chromatin prepared from SVWI38 and CaSki cells. DNA was immunoprecipitated using α-E2F2 or α-Dp1 antibodies and used in a PCR with primers spanning the E2F sites in the Kpnβ1 and Kpnα2 promoters (Fig. 4A, B). Positive amplification in both SVWI38 and CaSki cell lines confirmed an association between E2F2/Dp1 and the E2F sites in both the Kpnβ1 and Kpnα2 promoters (Fig. 4A, B).

Further, to determine whether the binding of E2F/Dp1 to the Kpnβ1 and Kpnα2 promoters was responsible for the elevated levels of both Kpnβ1 and Kpnα2 proteins *in vivo*, E2F activity was inhibited by silencing the expression of Dp1, without which E2F can no longer function. Kpnβ1 and Kpnα2 protein levels were measured by Western Blot analysis after Dp1 knock-down using siRNA, and were found to decrease after Dp1 inhibition (Fig. 4C). Kpnβ1 and Kpnα2 promoter activities were similarly repressed upon Dp1 inhibition (Fig. 4D, E).

### HPV E7 regulates Kpnβ1 and Kpnα2 promoter activities in cervical cancer cells via the repression of Rb

**Figure 5:**
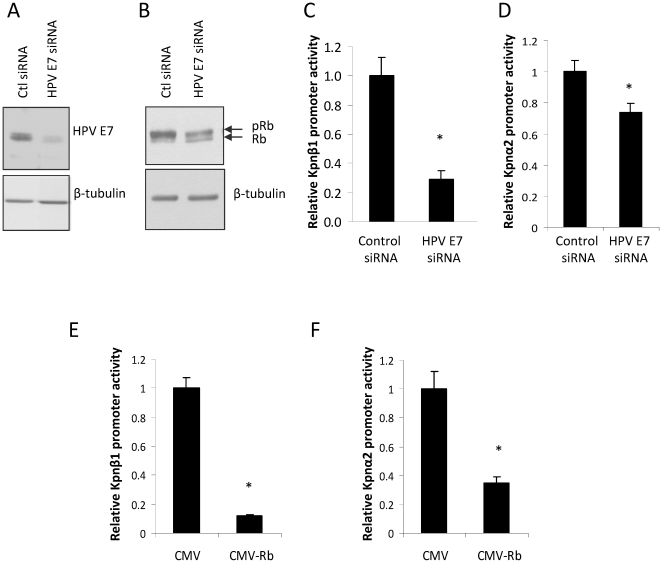
HPV E7 inhibition and Rb overexpression affects the activity of the Kpnβ1 and Kpnα2 promoters. A, B. HPV16 E7 siRNA was used to inhibit E7 expression in CaSki cells and Western blot analysis performed to analyse HPV E7 (A) and Rb (B) levels after HPV E7 knock-down. C, D. Kpnβ1 (C) and Kpnα2 (D) promoter activities were measured after E7 siRNA transfection, and were found to be significantly inhibited after HPV E7 inhibition. E, F. Rb was overexpressed in CaSki cells and Kpnβ1 (E) and Kpnα2 (F) promoter activities were found to be significantly inhibited. Results shown are the mean ± SE of experiments in quadruplicate performed at least two times (* p<0.05).

In cervical cancer cells the HPV E7 protein is known to bind the retinoblastoma (Rb) protein, leading to its phosphorylation and ultimately degradation. It is for this reason that E2F target genes are often overexpressed in cervical cancer, as E2F is no longer repressed by Rb and is thus free to activate its target genes. Hence, we hypothesised that the inhibition of HPV E7 in cervical cancer cells would lead to restored Rb function, causing reduced E2F activity, and thus diminished Kpnβ1 and Kpnα2 promoter activities. To test this hypothesis, CaSki cells were transfected with HPV16 E7 siRNA (Fig. 5A), and after confirming the restoration of Rb (indicated in Fig. 5B by a decrease in levels of phosphorylated Rb and an associated increased in unphosphorylated Rb), Kpnβ1 (−637 to +100) and Kpnα2 (−180 to +69) promoter activities were measured. Kpnβ1 and Kpnα2 promoter activities were significantly reduced in E7 knock-down cells, compared to control cells, albeit to different extents (Fig. 5C and D). To confirm that the decreased Kpnβ1 and Kpnα2 promoter activities were via the restoration of Rb function, Rb was overexpressed in CaSki cervical cancer cells and Kpnβ1 and Kpnα2 promoter activities measured. Results showed that Kpnβ1 and Kpnα2 promoter activities were both significantly reduced on overexpression of Rb (Fig. 5E and F). Together, these findings suggest that the high Kpnβ1 and Kpnα2 expression levels in cervical cancer cells is due in part to E7-mediated inhibition of Rb, and the consequent increased activation of the Kpnβ1 and Kpnα2 promoters by E2F.

## Discussion

In this study we cloned and analysed the Kpnβ1 and Kpnα2 promoters with an aim to identify potential regulatory mechanisms that could drive their high expression in cancer cells. This study is novel in that it is the first to our knowledge that describes E2F as an important transcriptional regulator of Kpnβ1 and Kpnα2 expression in cancer cells. E2F plays a role in a diverse range of cellular processes and has been reported to regulate the expression of genes involved in differentiation, development, proliferation and apoptosis [16]. It was previously reported that E2F plays a role in the regulation of the RanBP1 gene [17], a major partner of the Ran GTPase and integral to the nuclear transport process, and our study, describing E2F-regulation of Kpnβ1 and Kpnα2 nuclear importer proteins, further implicates E2F in the regulation of genes involved in nuclear transport.

E2F activity is controlled by the Rb pathway, whereby under normal circumstances Rb binding to E2F represses E2F activity, until the G1/S phase of the cell cycle, whereupon Rb becomes phosphorylated by cdk4/cyclin D complexes, and E2F is released to activate its target genes. However, E2F activity is often deregulated in cancer, as a result of the frequent disruption of the Rb pathway [18]–[21]. Rb mutations have been observed in a wide spectrum of tumours, including osteosarcomas, small cell lung carcinomas, breast carcinomas, and others. Moreover, in many cancers the p16^INK4a^ cdk inhibitor is mutated, or its function is loss. This leads to elevated cdk4/cyclin D activity, resulting in increased Rb phosphorylation and thus E2F accumulation. Finally, deregulated expression and/or amplification of cyclin D and cdk4 genes have also been observed in many cancers. This leads to an increased level of cdk4/cyclin D activity and thus similarly deregulation of the E2F pathway [21]. In cervical cancer, the inactivation of the E2F repressor, Rb, by HPV E7, results in increased E2F activity. As a result of frequent disruption of the pathway that regulates E2F function, E2F target genes are often overexpressed in cancer cells due to enhanced E2F transactivation [22].

We show that overexpresion of Kpnβ1 and Kpnα2 in cancer is due to, in part, increased activation of their promoters by E2F. A murine study by Ishida et al. [23] identified a potential role for E2F in the regulation of Kpnα2 gene expression during the cell cycle. Our study extends this finding and shows a role for E2F in the regulation of human Kpnα2, via a specific E2F site located at position −34 to −27 of the Kpnα2 promoter. This E2F site appears essential for both basal and activated Kpnα2 gene expression. In addition, we identified three functional E2F sites in the Kpnβ1 promoter that appear to act in concert to regulate Kpnβ1 expression. It has been reported that multiple E2F sites can exist in the promoter region of a gene, and that these sites often co-operate to regulate gene expression [24].

The finding that Kpnβ1 and Kpnα2 genes are regulated by E2F suggests that they are regulated in a cell cycle-dependent manner. It has previously been reported that the nuclear import of karyopherin α/β-substrates is more efficient at certain stages of the cell cycle [25], in agreement with our data showing E2F-regulation of Kpnβ1 and Kpnα2. Overall, our findings suggest that the deregulated activity of E2F in cancer cells causes increased activation of the Kpnβ1 and Kpnα2 promoters, leading to elevated levels of these proteins, and ultimately impacting the cancer phenotype.

## Materials and Methods

### Cell culture

WI38 normal lung fibroblasts, SVWI38 transformed WI38 fibroblasts, and CaSki cervical cancer cells were obtained from the American Type Culture Collection (ATCC) (Rockville, MD, USA). Cells were maintained in Dulbecco's Modified Eagle's Medium (DMEM) supplemented with penicillin (100 U/ml), streptomycin (100 µg/ml) and 10% Fetal Bovine Serum (FBS) (Gibco, Paisley, Scotland). All cells were cultured at 37°C in a humidified atmosphere of 5% CO_2_.

### Protein harvest and Western Blot analysis

Cells in culture were grown to 80% confluency and lysed on ice in RIPA buffer (10 mM Tris-Cl, pH 7.4, 150 mM NaCl, 1% deoxycholate, 0.1% SDS, 1% Triton X-100, 1 X Complete Protease Inhibitor Cocktail (Roche, Basel, Switzerland)). Western Blot analyses were performed using the rabbit anti-Kpnβ1 (H-300) (sc-11367, Santa Cruz Biotechnology, Santa Cruz, CA, USA), goat anti-Kpnα2 (C-20) (sc-6917), rabbit anti-Dp1 (K-20) (sc-610), mouse anti-HPV16 E7 (ED17) (sc-6981), mouse anti-Rb (4H1) (#9309, Cell Signaling), and rabbit anti-β-tubulin (H-235) (sc-9104, Santa Cruz Biotechnology) antibodies.

### PCR amplification of the Kpnβ1 (−2013 to +100) promoter

Primers were designed that would amplify the region from −2013 to +100 of the Kpnβ1 gene (GenBank Accession Number: NC_000017): Kpnβ1F 5′ AGGCTAGCCCAGCTACATCCAATTACCC 3′ and Kpnβ1R 5′ AG**AAGCTT**CTGGGGGCAGCTCAGA 3′ (NheI site is underlined and HindIII site is in bold), and −1992 to +69 of the Kpnα2 gene (GenBank Accession Number: NC_000017): Kpnα2F 5′ AGTT*CCCGGG*GCAAAAGAAACTCAACTTGGC 3′ and Kpnα2R 5′ AG**AAGCTT**GGGAATCAGCGGCTGTAG 3′ (SmaI site is italicised and HindIII site is in bold). PCR was performed using 100 ng normal blood DNA as template, and the high fidelity Expand Plus DNA Polymerase (Roche). 5% DMSO was included in the PCR for Kpnβ1 to improve specificity. Promoter PCR products were subcloned into the vector pGEM-T Easy (Promega) and the Kpnβ1 (−2013 to +100) fragment excised with NheI and HindIII restriction enzymes, and the Kpnα2 (−1992 to +69) fragment excised with SmaI and HindIII restriction enzymes, for subcloning into the luciferase reporter plasmid, pGL3 Basic vector (Promega). There were difficulties with the SmaI digest, hence SacI was used instead, as a SacI site was present at position −1900 of the Kpnα2 promoter. Subcloning into pGL3 Basic placed the Kpnβ1 (−2013 to +100) and Kpnα2 (−1990 to +69) promoter fragments upstream of the promoter-less luciferase gene for promoter activity analysis. Constructs were verified by sequencing using the UCT Human Genetics Sequencing Unit.

### Generation of Kpnβ1 and Kpnα2 promoter deletion constructs

Promoter deletion constructs for Kpnβ1 were generated using either restriction sites common to both the cloned promoter fragment and the pGL3-Basic multiple cloning site (KpnI for the −637 to +100 construct; SacI for the −271 to +100 construct), or gene-specific forward primers (5′ AGGCTAGCGCCGTCAGGAGAGCTTGA 3′ for the −861 to +100 construct; 5′ AGGCTAGCAGCGCCTGGCCAGTTAG 3′ for the −108 to +100 construct; NheI restriction site is underlined) and the Kpnβ1 R primer. The −180 to +69 deletion construct for Kpnα2 was generated using NruI and SacI restriction enzymes, which released the −1900 to −180 fragment, while the −24 to +69 deletion construct was generated using SwaI and SacI restriction enzymes, which released the −1900 to −24 fragment.

### Luciferase assays

100 ng of each promoter construct was transfected into 30 000 CaSki cervical cancer cells/well of a 24-well plate, using 0.3 µl Transfectin (Bio-Rad). To normalise for transfection efficiency the cells were co-transfected with 10 ng of the pRL-TK plasmid (that encodes Renilla luciferase). Total cell lysates were prepared from cells 24 hr post-transfection using 1 X Passive Lysis Buffer (Promega) and firefly luciferase activity was assayed using the Dual Luciferase Kit (Promega). Luminescence was monitored using the Glomax 96 microplate luminometer (Promega). Activity was normalised to the Renilla luciferase activity from pRL-TK in the same extract.

### Site-directed mutagenesis of putative E2F sites

Mutations in the E2F binding sites of the Kpnβ1 promoter were prepared by site-directed mutagenesis, using mutagenic primers: E2Fa: F: 5′ CGCTCAGGCCCGCtAgcACCTCGCGCAACAC 3′; E2Fa: R: 5′ GTGTTGCGCGAGGTgcTaGCGGGCCTGAGCG 3′; E2Fb: F: 5′ GAACCCAGCTCCGCCTCTTgCtaGcGGCGTCCCATCGTGCCCC 3′; E2Fb: R: 5′ GGGGCACGATGGGACGCCgCtaGcAAGAGGCGGAGCTGGGTTC 3′; E2Fc: F: 5′ GCTCCTAGGGTTGCtaGcCACCTCCCGCCTTCC 3′; E2Fc: R: 5′ GGAAGGCGGGAGGTGgCtaGCAACCCTAGGAGC 3′; E2Fd: F: 5′ CGGGCACCTCCCGCCTgCtaGCGCCCCGACCCCGAAC 3′; E2Fd: R: 5′ GTTCGGGGTCGGGGCGCtaGcAGGCGGGAGGTGCCCG 3′; E2Fe: F: 5′ CTCCTCTCCTTAGTTCTCgCtaGCACCCTATCCTATCAAC 3′; E2Fe: R: 5′ GTTGATAGGATAGGGTGCtaGcGAGAACTAAGGAGAGGAG 3′ (mutated bases are indicated in lowercase; NheI restriction site is underlined). Mutation of the E2F binding site in the Kpnα2 promoter was carried out using mutagenic primers: F 5′ CAATCGGAATGCGGAGTCgCtaGcAAATTTAAATCGCGCCGGGC 3′ and R 5′ GCCCGGCGCGATTTAAATTTgCtaGcGACTCCGCATTCCGATTG 3′. PCR was performed using the following conditions: 95°C for 30 seconds, followed by 18 cycles of 95°C for 30 seconds, 61°C for 1 minute, and 72°C for 6 minutes, followed by a final extension step of 72°C for 20 minutes. Following PCR amplification, 10 U DpnI (Promega) was added and digestion carried out at 37°C for 90 minutes, before transformation into JM109 highly competent cells (Promega). Restriction digestion analysis was used to identify clones carrying the mutation.

### Chromatin immunoprecipitation (ChIP) assay

Cells were grown to approximately 90% confluency and protein-DNA complexes cross-linked with 1% formaldehyde for 10 minutes, followed by the addition of 0.125 M Glycine, pH 2.5. Cells were harvested, lysed in lysis buffer (1% SDS, 5 mM EDTA, 50 mM Tris-Cl, pH 8.1, 1 X Complete Protease Inhibitor (Roche)), and sonicated to lengths of between 400 and 1000 bp. Cell lysates were diluted in dilution buffer (1% Triton X-100, 2 mM EDTA, 150 mM NaCl, 20 mM Tris-Cl, pH 8.1, 1 X Complete Protease Inhibitor) and then precleared with protein-A agarose beads (Merck, NJ, USA) for 2 hr. Beads had been previously blocked in 100 µg/ml salmon sperm DNA and 5% BSA. Chromatin was incubated with 2 µg antibody (α-E2F 2 (C-20 X, sc-633 X, Santa Cruz Biotechnology); α-DP1 (K-20, sc-610, Santa Cruz Biotechnology); or no antibody negative control) at 4°C overnight. Protein-A agarose beads were added for a further 2 hr at 4°C, and immunocomplexes bound by the beads recovered by centrifugation and washed twice sequentially in TSE I (0.1% SDS, 1% Triton X-100, 2 mM EDTA, 20 mM Tris-Cl, pH 8.1, 150 mM NaCl), TSE II (0.1% SDS, 1% Triton X-100, 2 mM EDTA, 20 mM Tris-Cl, pH 8.1, 500 mM NaCl), Buffer III (0.25 M LiCl, 1% NP-40, 1% Sodium Deoxycholate, 1 mM EDTA, 10 mM Tris-Cl, pH 8.1) and TE, pH 7.4. Bound material was eluted using elution buffer (1% SDS, 0.1 M NaHCO_3_) at room temperature for 10 minutes, and the input and eluted samples heated at 65°C overnight to reverse the formaldehyde cross-links. DNA was purified and used for real-time PCR, using primers designed to span the E2F binding sites (Kpnβ1 F: 5′ GCCATTAAAGCAGCTTCCTG 3′; Kpnβ1 R: 5′ CGGCAACCCTAGGAGC 3′; Kpnα2 F: 5′ CCCCAAGCCCTATGACTC 3′; Kpnα2 R: 5′ GGGAATCAGCGGCTGTAG 3′). These primers amplified a 199 bp and 204 bp product, respectively.

### siRNA transfection

For the effect of Dp1 siRNA on Kpnβ1 and Kpnα2 protein levels, 120 000 CaSki cells were plated in 35 mm dishes and transfected with 20 nM control siRNA-A (sc-37007, Santa Cruz Biotechnology) or Dp1 siRNA (sc-37813) for 72 hours, after which protein was harvested using RIPA buffer and Western Blot analysis performed.

For the effect of HPV E7 siRNA on Kpnβ1 and Kpnα2 promoter activities, cells were transfected with 20 nM HPV16 E7 siRNA. E7 siRNA was designed as described by Tang et al., 2006 [26]. RNA oligos were synthesised by Ella Biotech GmBH (Germany) and annealed in a reaction containing 30 µl each RNA oligo (50 µM) and 15 µl annealing buffer (100 mM Potassium acetate, 20 mM Hepes, pH 7.4, 2 mM Magnesium acetate) at 90°C for 1 minute, followed by 37°C for 1 hour, to give a final siRNA concentration of 20 µM. 24 hours after siRNA transfection cells were trypsinised and replated in 24-well plates, at 40 000 cells/well. 24 hours later, cells were co-transfected with 50 ng Kpnβ1 (−637/+100) or Kpnα2 (−180/+69) promoter constructs and 5 ng pRL-TK plasmid. Luciferase activity was measured 72 hours after siRNA transfection and expressed relative to Renilla luciferase in the same extract.

### Rb overexpression

To assay for the effect of Rb overexpression on Kpnβ1 and Kpnα2 promoter activities, cells were co-transfected with 100 ng pCMV or RcCMV/Rb (Addgene plasmid 1763, provided by Bob Weinberg), 50 ng promoter construct and 5 ng Renilla, using 0.45 ul Transfectin per well. Cells were incubated for 48 hours after transfection, and luciferase assays performed.

### Statistical analysis

Experiments were performed in triplicate or quadruplicate and are represented as the mean ± standard error of the mean (SEM). For group comparisons the Student's *t*-test was used, where two-tailed paired tests were used and a p-value of less that 0.05 considered statistically significant.
